# E-Mental Health Innovations for Aboriginal and Torres Strait Islander Australians: A Qualitative Study of Implementation Needs in Health Services

**DOI:** 10.2196/mental.5837

**Published:** 2016-09-19

**Authors:** Stefanie Puszka, Kylie M Dingwall, Michelle Sweet, Tricia Nagel

**Affiliations:** ^1^ Menzies School of Health Research Charles Darwin University Casuarina , NT Australia; ^2^ Menzies School of Health Research Charles Darwin University Alice Springs Australia

**Keywords:** eHealth, Indigenous health services, mental health services, diffusion of innovation, culturally appropriate technology

## Abstract

**Background:**

Electronic mental health (e-mental health) interventions offer effective, easily accessible, and cost effective treatment and support for mental illness and well-being concerns. However, e-mental health approaches have not been well utilized by health services to date and little is known about their implementation in practice, particularly in diverse contexts and communities.

**Objective:**

This study aims to understand stakeholder perspectives on the requirements for implementing e-mental health approaches in regional and remote health services for Indigenous Australians.

**Methods:**

Qualitative interviews were conducted with 32 managers, directors, chief executive officers (CEOs), and senior practitioners of mental health, well-being, alcohol and other drug and chronic disease services.

**Results:**

The implementation of e-mental health approaches in this context is likely to be influenced by characteristics related to the adopter (practitioner skill and knowledge, client characteristics, communication barriers), the innovation (engaging and supportive approach, culturally appropriate design, evidence base, data capture, professional development opportunities), and organizational systems (innovation-systems fit, implementation planning, investment).

**Conclusions:**

There is potential for e-mental health approaches to address mental illness and poor social and emotional well-being amongst Indigenous people and to advance their quality of care. Health service stakeholders reported that e-mental health interventions are likely to be most effective when used to support or extend existing health services, including elements of client-driven and practitioner-supported use. Potential solutions to obstacles for integration of e-mental health approaches into practice were proposed including practitioner training, appropriate tool design using a consultative approach, internal organizational directives and support structures, adaptations to existing systems and policies, implementation planning and organizational and government investment.

## Introduction

### E-Mental Health Services for Indigenous Australians

The role of digital technologies in addressing pervasive mental illness in the community has been explored over the past 15 years. The accessibility of new technologies to those with mental health concerns at any time or location, at low or no cost, and with a degree of anonymity has the potential to expand access to mental health services. Electronic mental health (e-mental health) interventions have emerged in response to this opportunity but their potential has not yet been fully realized.

E-mental health approaches provide treatment and support to people with mental health concerns through telephone, mobile phone, computer, and online applications, and range from the provision of health information, peer support services, virtual applications, and games through to real-time interaction with practitioners. They may be client-driven, practitioner-supported or involve a combination of support and self-driven use. There is growing evidence for the efficacy and cost effectiveness of e-mental health approaches [1,2].

E-mental health may provide a novel opportunity for improved health and well-being for Aboriginal and Torres Strait Islander (Indigenous) Australians. The potential adoption of e-mental health approaches amongst Indigenous people, however, has not yet been well explored [3]. Although diverse in life experiences, circumstances, and histories, Indigenous Australians display very high overall rates of psychological distress [4] and suicide [5], which may result from colonization, dislocation from country and loss of identity, previous government policies of assimilation and child removal, marginalization from mainstream society, institutionalized racism and poor education and employment outcomes, and intergenerational trauma [6,7]. Furthermore, Indigenous Australians do not access mental health services at rates commensurate with their burden of disease [4]. Often living in rural and remote areas, access to appropriate services for Indigenous people may be limited by lack of availability in addition to other factors such as the cultural inappropriateness of services and stigma associated with seeking treatment [8].

The Australian Government supports the use of e-mental health approaches by consumers and health services, including within Indigenous populations, through its National e-Mental Health Strategy. A key component is an e-mental health training and support service for practitioners working in primary health care known as e-Mental Health in Practice (eMHPrac). The present research was conducted within the Indigenous stream of eMHPrac in the Northern Territory.

### Current Use of E-Mental Health

Despite the demonstrated potential, e-mental health approaches have not been well utilized in health services [9] and there is a general dearth of research into implementation and use within service settings [10,11]. While evidence-based treatments are proliferating [12], the number of evidence-based implementation strategies remains few [13]. Many theoretical frameworks seek to describe the dynamic process of the implementation of innovations. These various frameworks, strongly influenced by Rogers, seek to describe the findings that health care innovations are implemented more successfully when certain conditions are favorable [14]. Greenhalgh et al sought to combine this diverse literature into a unifying model of the diffusion of innovations in health care organizations which included characteristics of the user system, the outer context, the innovation itself, and the adopters within the organization [15].

Over a decade ago, Whitfield and Williams found that the most significant impediments to use of e-mental health in health services were a lack of skills amongst practitioners, unclear guidelines for use, and negative perceptions about e-mental health (such as confidentiality concerns, lack of confidence in use, and perceptions of e-mental health as inferior to face-to-face therapy) [16]. However, in a more recent review of the implementation of electronic innovations in youth health services, Montague and colleagues found that a lack of time and resources, poor information technology (IT) skills amongst staff, and technical problems provided the greatest encumbrances, while negative perceptions of e-mental health amongst staff and clients did not significantly inhibit use. [11]

In a recent systematic review of e-mental health use by Australian consumers and practitioners, Meurk and colleagues described facilitators of e-mental health use that included therapist support for innovations, mental health literacy, convenience factors, integration into existing care, and cultural appropriateness [17]. Barriers included lack of awareness of e-mental health amongst clients and practitioners and lack of e-mental health training for practitioners. Several factors were reported to act as both facilitators and barriers across different studies (perceived anonymity of e-mental health, stigma associated with help-seeking, gender, rural residence, concerns about privacy, preferences for self-help), suggesting that contextual factors are likely to have a large influence on results. Meurk and colleagues also reported a need for further research exploring the appropriateness of e-mental health with different population groups. The current study aims to understand stakeholder perspectives on the requirements for implementation of e-mental health approaches in regional and remote health services for Indigenous Australians.

## Methods

### Data Collection

Semi-structured, qualitative interviews were conducted with 32 stakeholders to explore current and potential use of e-mental health approaches with Indigenous clients. Findings will be used within a broader formative evaluation framework [18] of training and support provided by the eMHPrac support service in the Northern Territory of Australia.

We acknowledge the subjectivity of our position as researchers within the eMHPrac collaboration and as developers of an e-mental health tool (the AIMhi Stay Strong App); however, throughout we have sought to render our position explicit and to reflect on how our experiences may have shaped our findings while considering other perspectives [19].

Ethics approval was granted by all relevant ethics committees (ref #HREC 12-1881 and #CAHREC 12-100) including an Aboriginal sub-committee.

### Recruitment

Interviews were conducted between December 2013 and March 2015 by SP, MS, and KD with managers, directors, chief executive officers (CEOs), and senior practitioners (eg, clinical supervisors including general practitioners, nurses and psychologists) of mental health, well-being, alcohol and other drug and other services (primarily chronic disease) working with Indigenous people in the Northern Territory of Australia. Participating organizations were either government health services or other non-profit, predominantly publicly-funded services: non-government organizations (NGOs) and Aboriginal community controlled health organizations (ACCHOs) offering a broad range of services and programs (eg, counseling, alcohol and drug rehabilitation, social support and primary care) (Table 1).

**Table 1:** Participant roles, organization types, and service types (N=32).

Participant		n (%)
**Role**		
	Manager/CEO/director	21 (66)
	Practitioner	11 (34)
**Organization type**		
	Government health service	12 (37)
	Aboriginal community controlled health organization	6 (19)
	Non-government organization	14 (44)
**Service type**		
	Alcohol and other drug service	9 (28)
	Mental health service	13 (41)
	Social and emotional well-being service	2 (6)
Other		8 (25)

A semi-structured interview guide was developed informed by literature, our previous research findings, and discussion among the research team. The guide covered knowledge of and attitude toward e-mental health resources (eg, Do you use or know of any e-mental health resources/strategies? If yes, which ones?) and a range of perceived implementation challenges and facilitators (eg, Can you identify any barriers to the e-mental health approaches in your practice and potential solutions to these barriers, for example, policy, staff availability, staff turnover, training?). The following description of e-mental health was provided to participants at the commencement of interviews:

e-Mental health services provide treatment and support to people with mental health disorders through telephone, mobile phone, computer and online applications and range from the provision of health information, peer support services, virtual applications and games through to real-time interaction with clinicians trained to assist people experiencing mental health issues.

Participants were initially approached based on personal and professional networks and knowledge of the sector prior to participating in e-mental health training. Snowball and maximum variance sampling techniques were used to collect data from a broad range of organization and service types and to ensure inclusion of participants from outside our networks. Although interviews explored e-mental health in general, all participants had been provided with information about the AIMhi Stay Strong App prior to the interview and for many, this was the e-mental health tool that they were most familiar with. As a result many of the responses were focused on the use of e-mental health through mobile devices.

Some stakeholders (60%, 19/32) participated in group interviews with up to four colleagues while others (41%, 13/32) were interviewed individually. Decisions about interview format and setting were determined by participant preference and convenience. Interviews were recorded, transcribed verbatim and checked against audio recordings.

### Analysis

An adapted grounded theory methodology was used to construct localized knowledge about the use of e-mental health in health services for Indigenous Australians from the data [20]. Our analysis oscillated between deductive and inductive approaches. Data were broken down into discrete parts and compared and contrasted with remaining data, and were put back together in new categories, making connections between categories involving conditions, context and consequences following Strauss and Corbin’s method [20]. All authors immersed themselves in the data by reading and re-reading transcripts. Authors each developed codes and then arrived at a single set of axial codes (adopters, the innovation, and user system) and sub-codes within each through group consensus following reference to the model of the diffusion of innovations in healthcare organizations proposed by Greenhalgh et al [15]. The consensus codes were then applied to the data and as the key results in each code were initially summarized, codes were refined to reduce duplication and more clearly present findings. NVIVO software was used to support storage and coding of transcripts.

## Results

### Adopters

**Figure 1:**
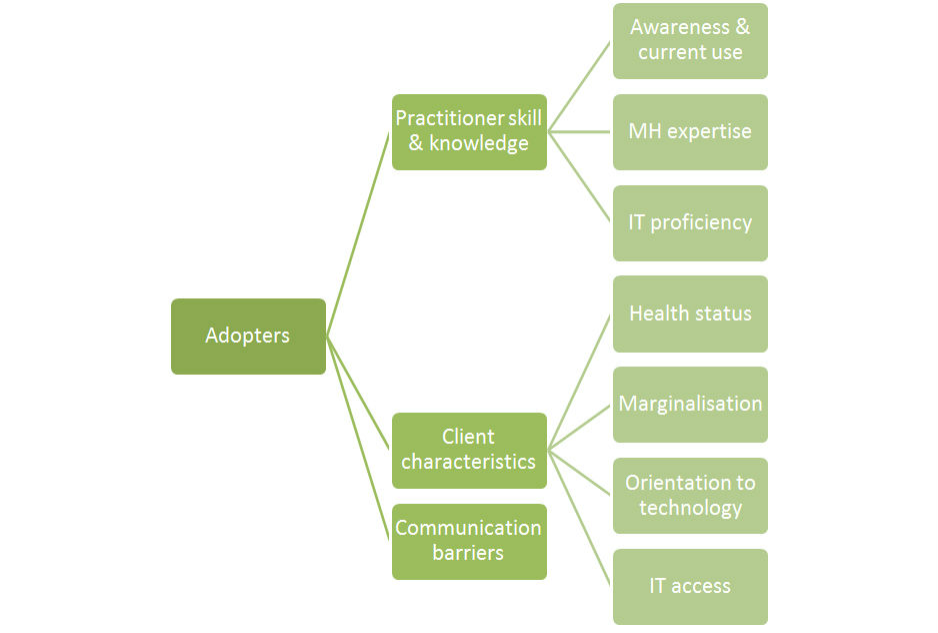
Adopter (practitioner and client) factors impacting use of e-mental health approaches.

Participants described the skills, experience, and personal attributes of practitioners and clients that would potentially influence the use of e-mental health in practice (Figure 1).

### Practitioner Skill and Knowledge

Potential e-mental health adopters among health practitioners were defined as representing a diverse cross-section of the community. Participants perceived that different levels of mental health training and proficiency with IT and generally low levels of knowledge and awareness of e-mental health present challenges to implementation of e-mental health approaches in practice.

#### Awareness and Current Use of E-Mental Health

Lack of awareness was a key impediment to implementation. Very few participants had used e-mental health tools and some were not familiar with the concept of e-mental health. For example, one ACCHO manager commented: “I think this e-based, what do you call it, this e-mental health approach is a new concept, can I say for the Aboriginal health sector.”

Of those who had some awareness of e-mental health, the most common tools mentioned were online cognitive behavioral therapy (CBT) programs and Internet- or phone-based counseling services in general, and the AIMhi Stay Strong App and beyondblue website specifically. Only one participant reported use of e-mental health services in practice through referral of clients to mindfulness apps. No participants reported previous exposure to e-mental health training.

#### Mental Health Expertise

Lack of mental health expertise amongst health practitioners was perceived to impact upon e-mental health uptake. Participants from a range of service types (mental health, alcohol and other drugs, community services) noted that some staff did not have formal qualifications in mental health.

Some of them haven’t done a basic Cert IV in mental health, so some of them have studied assessment but some of them haven’t. They haven’t got that sort of grounding.

Manager, NGO

One participant expressed concern about risks in her staff using e-mental health tools which may induce them to make decisions about treatment which they were not qualified for.

#### Information Technology Proficiency

IT skills were identified as a key influence in e-mental health implementation. Participants described their colleagues as exhibiting varied levels of skill and comfort in using technology. The use of mobile devices such as tablets was new for many.

There’s probably one or two [staff] that are proficient, can use a computer, the rest can’t. They barely can send an email… They just freak out at the thought of having a tablet.

Manager, ACCHO

We are increasingly becoming better at using new technologies.

Manager, ACCHO

Age was reportedly an important determinant of familiarity and comfort with IT amongst health practitioners.

### Client Characteristics

Health and socio-economic status emerged as client factors which may influence their ability or willingness to use e-mental health tools.

#### Poor Health Status and Well-Being Challenges

Poor health status and significant well-being challenges with high levels of comorbidity were thought to negatively impact on the suitability of e-mental health tools for some clients. One government health service manager said: “Often people with serious mental illness or with poor literacy, a lot of that stuff’s still not suitable for them.”

These factors were described as impacting upon clients’ concentration and their ability to sit through a session with a practitioner to complete an e-mental health tool.

There’s a lot of young people, whether they’re diagnosed or not, are ADH or oppositional or any of that crap. To try to get them to sit in one place for more than two seconds is quite difficult and a lot of these issues have been compounded by fetal alcohol syndrome, compounded by other primary health issues, compounded by substance abuse.

Manager, NGO

Poor concentration was particularly attributed to younger clients, however in the case of young clients receiving renal dialysis, using mobile devices was seen as an antidote to boredom.

#### Marginalization

Despite the need for mental health services, it was suggested that lack of engagement may impede clients from seeking treatment, including through e-mental health approaches. Poor health and well-being were described as being compounded by marginalization from society and the grief and trauma associated with events in some clients’ communities such as suicide and conflict. A large number of participants described some of their clients as disengaged from the health care system, not well involved in decisions about their own health care or not fully participating in therapy, frequently missing appointments. A manager at a NGO said: “It’s generational, it’s the times, it’s welfare dependency, it’s the lack of an appropriate educational system, it’s all that and what’s work, why do you want me to work, you know that sort of despair that comes with addiction.”

#### Orientation Towards Technology

Stakeholders described their clients as generally being positively oriented towards new technologies: “Young people are really into iPads and electronic equipment” (Practitioner, government health service).

Electronic media were described generally as an important component of modern Indigenous youth culture and identity and most agreed that e-mental health tools would be particularly appropriate for this demographic. However, a small number of participants discussed the high level of client motivation needed for them to access some e-mental health tools independently.

A tool like that [AIMhi Stay Strong App] you need motivation to do it independently, so it will be a great tool to use with a worker or with someone you’re sort of walking through things. Yeah, but if you just put it out there as an output, I don’t know how many people would access it.

Coordinator, NGO

#### Information Technology Access

Client access to IT was thought to be an important determinant of use. While most reported high rates of mobile phone ownership, including in remote communities, rates of smart phone or tablet ownership were thought to be lower and it was noted that some remote communities still did not have Internet connection.

mobile phone concentration’s very high in the bush …

Manager, government health service

… in a lot of communities… there is mobile access, which still isn’t all the communities in the western desert by any stretch….

Manager, ACCHO

Although financial constraints were not specifically raised, one participant suggested that health services could subsidize the cost of e-mental health tools for clients.

### Communication Barriers

Potential communication barriers between staff and clients were discussed as factors influencing the effective use of e-mental health in health services. Practitioners and clients may not speak English as a first language or may have limited English literacy, presenting possible impediments to accessing mental health services, including e-mental health.

Our staff vary from people with uni degrees and used to working in hospitals and doing questionnaires and stuff to people who are very practically based and we have 35% Indigenous employees with a range of experience, tertiary and literacy and numeracy skills…

Manager, ACCHO

English is not the first language for most of them [Indigenous clients].

Senior practitioner, government health service

### The Innovation

**Figure 2:**
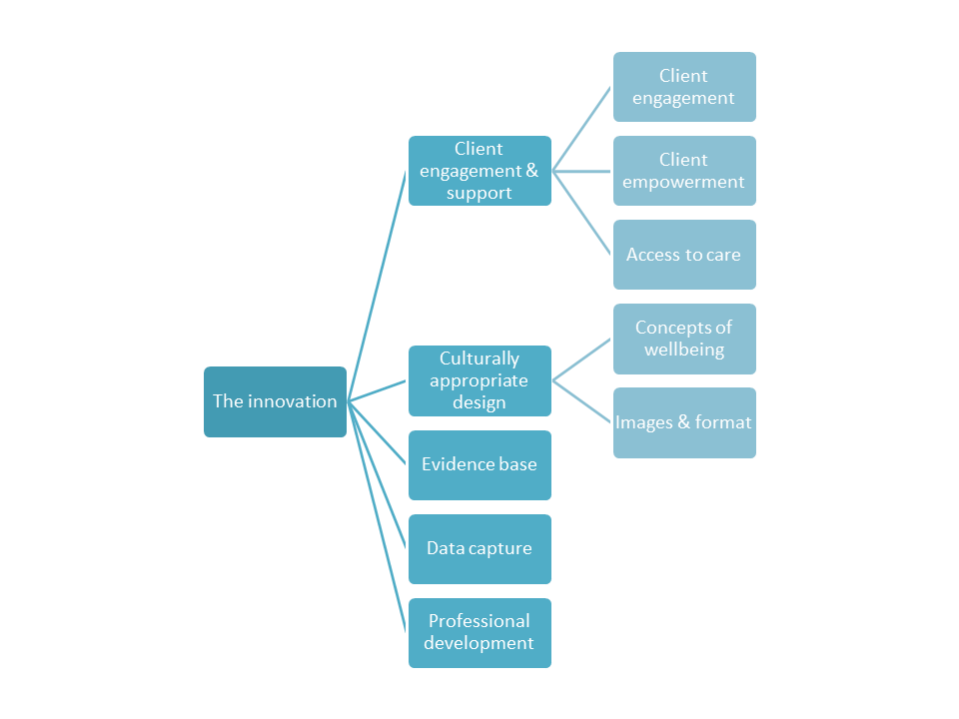
Innovation-related factors impacting use of e-mental health approaches.

Particular aspects or design features of e-mental health tools which may influence use were explored (Figure 2).

### Client Engagement and Support

#### Client Engagement

E-mental health tools which support engagement between clients and practitioners in mental health services were viewed by most participants more favorably, especially those with the potential to lead to more open dialogue through a less direct approach to sensitive issues.

it will make it easier to engage with some clients and again, I don’t know whether I’m just a victim to stereotypes, but you think that young people who don’t necessarily have the willingness or the language to kind of engage in those conversations in just a face to face chat…

Coordinator, ACCHO

...it allows people to focus on an indirect object, which would make that relationship building easier. So any elements of shame and embarrassment could be mediated to some level by the use of a tool that is introduced into that interaction.

Manager, NGO

However, two participants saw potential for e-mental health tools used within a client session to intrude on the therapeutic relationship if tools were not understood by the client or if they interrupted a conversation, suggesting that introducing an electronic based tool to a client session would require some judgment and skill.

The optimism amongst participants in the potential for e-mental health tools to strengthen client engagement appeared to be limited to practitioner-supported tools. A small number of participants described client-driven tools as potentially disengaging and alienating clients. One participant commented: “It’s around relationship stuff, so that’s why, like that’s awesome because you can sit there and be with the person and use it together and still have that relationship with the young person, whereas sometimes it’s hard to establish that if they’re working with a computer screen...” (Coordinator, NGO).

#### Client Empowerment

E-mental health tools which empower clients in the recording of their information were well received. Practitioner-supported e-mental health tools such as the AIMhi Stay Strong App were contrasted favorably with pen and paper approaches to collecting client information. Several participants described how pen and paper approaches could be disempowering experiences for clients due to low literacy and as reminders of intergenerational institutionalization.

[The AIMhi Stay Strong App] has a collaborative approach that the information that is gathered is gathered with full awareness and permission of the participant.

Manager, ACCHO

it’s certainly a whole lot less scary … than this pen and paper because there’s things to push.

Manager, NGO

One participant discussed preferences for tools that enable both staff and clients to enter information during and between sessions.

#### Access to Care

Participants saw a role for e-mental health in providing additional support during or between client sessions and acting as a “soft entry point into services *”* (Coordinator, NGO). The convenience of accessing e-mental health at any time of day and from any location was thought to supplement, but not replace face to face client sessions: “…giving your client like an extra dose of therapy like working with them and then sending them away with stuff that they love to access” (Coordinator, NGO).

However, one participant drew attention to the risk of creating new inequalities in the access to care: “...for people who are better educated they’ll just take them to another level and the people who are less educated will get left further behind” (Manager, ACCHO).

### Culturally Appropriate Design

#### Concepts of Well-Being

The small number of participants who offered comment appeared to favor e-mental health tools that adopted a cross-cultural approach by encompassing or acknowledging Indigenous concepts of mental health. Participants tended to favor a holistic and less medicalized approach to mental health concerns as represented by the broader term *social and emotional well-being*.

Aboriginal people don’t see themselves in those sorts of stigmatized ways [diagnosed with bipolar disorder, schizophrenia etc]. They mainly refer to them as people with social emotional well-being type...you know they prefer to have a much softer approach to that…. It’s more a subtle approach than a clinical approach.

Manager, ACCHO

#### Images and Format

Participants described the optimal overall design of e-mental health tools as being *easy to navigate* or *self-explanatory* to aid staff and clients who were not confident with technology. Translation into Indigenous languages or use of plain English was also thought to promote use. In addition, the use of audio prompts in Indigenous languages or plain English was recommended.

The use of *visual aids* to help explain complex mental health concepts and the inclusion of aesthetically engaging content was thought to be particularly important. The nature of desirable images and graphics were described as *friendly* and *non-threatening*, suggesting a need for sensitivity in graphic design. However, in reviewing the AIMhi Stay Strong App, one participant described the potential for some Indigenous clients to view the simple text and visual prompts as patronizing, suggesting a need to ensure simplifying content does not result in omitting information or adopting a disrespectful stance.

### Evidence Base

Participants confirmed that robust evidence of effectiveness would promote e-mental health implementation. Participants were generally unsure about the evidence base and effectiveness of e-mental health; however, there was optimism about its potential and consensus that more information and research was needed. Many called for more research, particularly within Indigenous populations: “We need to be aware of those apps, which ones are good, which ones are evidence based and which ones are crap” (Manager, ACCHO).

### Data Capture

There was a general perception of both risks and benefits in the data capture functions of e-mental health tools. In particular, the capacity to measure outcomes in a clear, easy to interpret format, potentially using an automated function, was an attractive feature for health services. The ability to document a broad client history, including family mapping and other information specific to an individual client, was also appreciated by participants when discussing the AIMhi Stay Strong App: “We want to empower people in local communities to be able to access information and to record their own stories” (Manager, ACCHO).

However, there was a concurrent concern about the security of confidential client data collected by e-mental health tools. Several participants perceived e-mental health to afford a lower level of security than existing electronic information systems, particularly when information was stored in mobile devices and transmitted via email: “I’ve always been concerned about electronic forms of client recording, which is really around confidentiality and the ease with which things can be copied and transferred to people” (Manager, NGO).

### Professional Development

Practitioner-supported e-mental health tools that adopt a structured approach or prompts for best practice care were perceived as providing professional development for practitioners. “The other beauty of the best e-health [tools], they can be quite a useful professional development tool for our staff, like you see some of these websites and then you think well they’ve got that right, my personal professional practice should at least match that” (Manager, ACCHO).

Prompts for health promotion messages, client engagement, identifying well-being markers and assessing client risk were appreciated.

### User System

**Figure 3:**
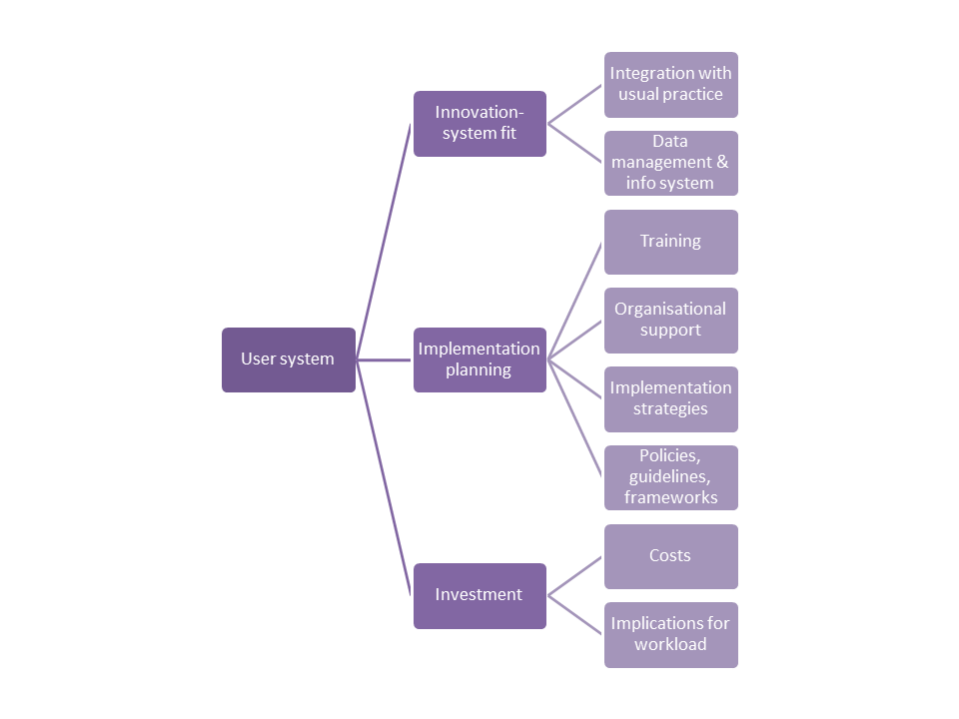
User system factors impacting use of e-mental health approaches.

The factors supporting or inhibiting adoption of e-mental health approaches within stakeholders’ service delivery settings were explored (Figure 3).

### Innovation-System Fit

#### Integration With Usual Practice

The degree to which e-mental health tools captured or integrated with various aspects of usual practice in client care was described as an important factor mediating use. Most participants suggested e-mental health tools would integrate successfully. Many saw a role for e-mental health tools such as the AIMhi Stay Strong App in an initial session with a new client, as an engagement tool to help build a therapeutic relationship, as an additional component of client assessment, or both. Many saw a potential for e-mental health to replace current care planning processes, or to integrate with existing care plans. A number of participants also discussed the potential for e-mental health tools to improve case management within their organizations: “… probably fitting it somehow into their mental health care plan, their chronic disease care plan would be the best way” (Manager, government health service).

Some participants saw e-mental health tools as having the potential to support health services in remote community settings. The flexibility and portability of mobile devices were described as a favorable feature supporting such work.

#### Data Management and Alignment With Information Systems

Participants generally saw potential for e-mental health tools to support client data management within organizations but that challenges in integrating tools within existing information systems may detract from use. E-mental health tools were seen to offer support in client data management across organizations through immediate recording and availability of electronic data, measurement of client outcomes, reporting of client data, and improving client ownership of records. A number of participant perspectives on data management were expressed in the context of outdated or poorly managed existing information management systems, including continued reliance on paper-based record systems: *“* It will improve our capacity for making sure documents don’t get lost…” (Manager, ACCHO).

While some participants saw e-mental health tools as providing a desirable addition to existing information management systems, others warned of the risks in failing to integrate e-mental health within existing systems, including the potential for information to be lost. Participants described a need for further technical work to ensure e-mental health tools were accessible within existing systems. They also described a need for data to be easily transferred between systems and clients to be easily identified across systems.

### Implementation Planning

#### Training

Participants differed in their views on the degree of change needed to implement e-mental health approaches into practice within health services and the steps required. However, almost all agreed that some staff training would be needed. Some participants added that staff in their organization would need other types of training in order to use e-mental health tools, including training in counseling skills, in using mobile devices, and in working in a cross-cultural context. A common theme amongst participants was the need to ensure sustainability of training programs and to address high staff turnover across the sector. Several participants recommended embedding e-mental health training in orientation processes for new staff.

#### Organizational Support

A number of participants described the importance of establishing supportive structures within organizations such as supervision, regular review, and helpdesk support in promoting implementation and treatment fidelity of e-mental health approaches: “You can have the training, but if you don’t have the supervision and the support behind it, it never gets done” (Manager, NGO).

Support and commitment from senior managers were also thought to be an important aspect of the change process which would facilitate greater levels of adoption of e-mental health approaches.

#### Implementation Strategies

A small number of participants discussed development of specific organizational implementation planning strategies. Aspects of the planning process described included introducing e-mental health initially in a pilot process, using a staged approach, determining which aspects of usual practice e-mental health will replace, decisions on how the use of e-mental health tools will be documented in client records, and establishing and reviewing implementation milestones. Only one participant mentioned a need to consult with staff and clients.

#### Policies, Guidelines, and Frameworks

Participants suggested that effective and ethical e-mental health use should be governed by organizational policies, guidelines, and frameworks. Some thought that existing clinical governance frameworks could provide appropriate guidance for managing potential risks to clients, responding to incidents, and providing clinical supervision, and that existing client consent policies would equally apply to provision of e-mental health treatment.

There were mixed opinions as to whether existing policies on appropriate use of technology and the secure storage and transmission of electronic data would need to be adapted to take account of e-mental health use: “I think that there needs to be a lot of rigor around...controls around how this gets moved and transferred” (Manager, NGO).

A small number of participants described existing policies as preventing access to e-mental health tools (eg, restricted Internet use) and mobile devices. However, others described a need for policies to mediate appropriate use of and access to mobile devices, suggesting policies needed to achieve a balance between access and risk management.

### Investment

#### Costs

The costs and expected benefits associated with e-mental health were a key consideration for participants in adopting e-mental health approaches within their organizations. Participants represented government departments or non-profit organizations and described financial constraints and uncertainty about future funding. The most common costs identified were in purchasing mobile devices and training staff. Although free training was offered within eMHPrac, there were associated costs in backfilling frontline staff and travel to be considered. The need to upgrade existing systems to enable utilization of e-mental health was also identified as a cost.

However, almost all participants planned to take advantage of the free training offered and to seek funding for mobile devices where not already available, suggesting that the costs involved were perceived as a worthwhile investment: “…I think that would be a cost benefit sort of situation equal…” (Manager, ACCHO).

Potential risks to the investment were discussed, such as the potential for mobile devices to be lost, damaged or stolen, and the possibility of hidden costs, for example in future upgrades to devices and e-mental health tools.

#### Implications for Workload

Some noted the potential for the initial investment in e-mental health to result in future savings for organizations through improved efficiency. Efficiency dividends were thought to arise from reducing the amount of staff time in writing client notes and the immediate transmission and availability of client data. However, others saw potential for e-mental health use to result in longer client sessions and thought the implementation process would increase staff workload initially.

## Discussion

### Principal Findings

There is strong potential for e-mental health approaches to complement existing mental health services for Indigenous people. However, several client, practitioner and system-level factors as well as the design of innovations will determine the level of perceived usefulness and ultimately the success of implementation efforts.

#### Adopters

Limited knowledge of e-mental health tools amongst health practitioners is a key obstacle to use. This finding accords with results from a recent eMHPrac training evaluation [21] and reports of poor implementation of e-mental health approaches across Australian health services, despite Australian developers playing a leading role in the development of e-mental health innovations internationally [22]. Similarly, a recent systematic review found that one of the key impediments to more widespread use included lack of awareness and knowledge about e-mental health amongst clients and practitioners [17]. Our findings support the conclusions drawn on the need for greater promotion and awareness-raising amongst practitioners.

Mental health training and expertise amongst health practitioners is another factor driving use of e-mental health tools. These findings are in accord with those of a study which aimed to identify contextual influences on integration of innovations in mental health services in both UK National Health Service and community settings. Brooks et al (2011) found that service provider skills and knowledge were among the top enablers to implementation [23]. This theme of adopter confidence is also reflected in the findings of Panzano and Roth who found that the decision to adopt an innovative mental health practice involves consideration of risk [24]. Their data suggest that early adopters see the risks associated with adopting as lower and potentially more manageable.

The Northern Territory mental health and well-being workforce is varied and often limited in terms of formal training or qualifications [25]. Some have found that e-mental health approaches can be implemented successfully by practitioners with little mental health training [26]. An evaluation of a one-day e-mental health training course provided through eMHPrac has shown that participants with diverse roles and professional backgrounds improved their knowledge and skills in e-mental health significantly post-training [21]. The availability of appropriately-targeted training in e-mental health will be a crucial component of e-mental health implementation; however, workforce factors also indicate a need earlier in the development phase for production of new tools which are evidence-based, easily understood, appropriately targeted and require little training.

For Indigenous clients, stakeholders expressed preferences for tools that involve a level of practitioner support (Multimedia Appendix 1). This accords with the oral traditions of Indigenous Australians and the emphasis often placed on personal relationships [27]. Furthermore, studies of online treatments (particularly Internet-based CBT) have shown the largest effect sizes when combined with practitioner support [28,29]. Nevertheless, some level of client-driven use was also supported by stakeholders who saw an opportunity for e-mental health to complement traditional approaches to treatment and support self-management.

Reynolds et al [30] offer a framework of e-mental health use in primary care, describing a continuum of therapist involvement from promotion to case management, coaching, integration into symptom-focused therapy, and integration into comprehensive treatment. Our findings suggest that e-mental health tools which can be integrated into face to face therapy or treatment, or tools that can be used in a case management or coaching scenario, where service providers offer emotional and technical support to clients to use e-mental health tools either within a face to face session or externally, were preferred.

#### The Innovation

Specific design of e-mental health tools to address the needs of Indigenous Australians will facilitate use. As others exploring the use of e-mental health innovations with Indigenous Australians have found, very few tools have been developed to address the needs of Indigenous people [3,31]. Appropriate tool design has been identified as a key facilitator of acceptance of e-mental health tools [9]. E-mental health tools that adopt a social and emotional well-being framework acknowledging the broader social determinants of mental health may have more capacity for including Indigenous concepts of mental health than biomedical models. Christie and Verran have also called for the development of tools for Indigenous Australians that adopt a holistic perspective of health and the health system, and with interactive features than enable development of shared understandings rather than only transferring information [32].

However, there are challenges in developing a tool which reflects Indigenous worldviews and understandings of mental health, life experiences, and conversational style, particularly in the context of an extremely diverse population. Developers must seek to both avoid oversimplifying concepts or presenting Indigenous perspectives in a tokenistic manner and yet also avoid rendering tools and concepts too complex. While design features such as images, language style and tone, and audio prompts can impact on ease of use and successful uptake [33], developers may also encounter intellectual property issues and sensitivities to the use of certain images, words or concepts in certain contexts [34]. As others investigating the development of e-mental health innovations designed for Indigenous Australians have found, there continues to be a need to ensure that content is localized and adapted to different regions, communities and languages through consultative, collaborative approaches [3,35].

The security of personal data stored in e-mental health tools was a significant concern for participants and provides an imperative for developers to address prior to the integration of tools into routine care. The potential for e-mental health tools to record highly sensitive information while providing the means for rapid transmission of information online is a key risk. Historical and ongoing government intervention in the lives of Indigenous people [36] and difficulties in de-identifying client information in small inter-related communities may further heighten security concerns amongst this population group. Although Povey and colleagues did not find that data security concerns significantly detracted from the appeal of e-mental health amongst Indigenous community members [3], other studies have reported perceived risks to confidentiality amongst practitioners and consumers [11,17], and it may be the case that health service managers in their duty of care to clients and experience in managing client data are more attuned to potential risks.

Those e-mental health resources which can be used in a client session and potentially improve professional practice through the use of prompts and reminders, and through better recording of client data and are accompanied by further training, are most attractive to health services. This need is recognized by the National e-Mental Health Strategy which is currently offering e-mental health training and implementation support through the eMHPrac support service [30].

Robust research evidence will generate greater confidence in the use of e-mental health tools in health services. This finding is in accord with our earlier investigation of user perspectives of a localized e-mental health tool [33]. Future research should consider the implementation context and practitioner fidelity to treatment to ensure that shifts in practice achieve the intended outcomes and are sustained over time.

#### User System

Health service organizational systems and processes with capacity to support e-mental health tools are needed. Current systems and supportive infrastructure such as Internet access and mobile devices were reported as often unavailable or outdated. Although Internet coverage in Australia is expanding, some remote communities remain without Internet connection while others continue to experience slow download speeds [37]. Poor Internet access has been reported as an impediment to e-mental health use in related studies [3,33]. A recently announced Digital Mental Health Gateway to support practitioner and client access e-mental health tools through a centralized portal as part of a new stepped-care system [38] may assist in connecting systems to e-mental health tools to some extent. Nevertheless, it is likely that service providers will need to adapt organizational systems and processes to some extent in order to integrate e-mental health approaches.

Other studies investigating implementation of mental health service innovations have similarly found the capacity and receptiveness of organizational systems to be pivotal in their success. Barnett et al identified a range of organizational factors either impeding or facilitating innovation: organizational receptiveness (including the fit between the innovation and the organizational ethos), available resources, as well as organizational capability to promote the innovation with other organizations [39]. Similarly Brooks et al found that resource limitations and lack of support from corporate departments such as HR and finance were key impediments to implementation [23].

Participants confirmed what is known from the diffusion of innovations literature: successful implementation of new innovations into service settings requires consultation, organizational support, support structures and lines of reporting established, planning, adaptation of current systems, supportive policies or guidelines and adequate resourcing [15]. As Montague and colleagues have found, the availability of e-mental health innovations has often preceded the development of organizational policies and protocols governing their use [11].

Implementation of e-mental health approaches requires investment in supportive infrastructure and workforce capacity building for many organizations, as well as investment in tools and devices themselves. The need for e-mental health supports and technical upgrades is recognized by the e-Mental Health Alliance [9]. However, these were costs that some stakeholders described as posing a burden on already stretched budgets. Further government investment in health service systems and supportive infrastructure is needed.

Addressing staff training needs and embedding training in existing structures is also required. The current piecemeal approach to e-mental health implementation with an apparent lack of staff consultation appears to be leading to a small number of “early adopters” and a large proportion of “laggards” [14]. In the absence of a whole-of-organization approach, including formal guidance in which aspects of current practice e-mental health will replace or complement and technical work to adapt or upgrade existing information management systems, there are risks that e-mental health could lead to duplication in the delivery of care and recording of client data or that initial changes to practice are not sustained.

#### Recommendations

Practitioners, developers, service providers and governments all have important contributions to make in realizing the opportunities presented by e-mental health (Textbox 1).

### Limitations

Participation in this study was restricted to service providers and we did not seek client, developer or community views directly. Nevertheless, the views expressed provide insight into current awareness of e-mental health and perceived potential for implementation in the health service settings in which the participants are expert. Indigenous community perspectives on the use of mobile applications for mental health have been explored elsewhere [3].

**Recommendations for practitioners, developers, and services to promote adoption of e-mental health approaches.**

Recommendations

- Practitioners
  - Seek advice from local consumer or community groups on the appropriateness of particular tools for particular client groups.
  - Choose e-mental health tools to suit the individual, considering client language and literacy, cultural factors, engagement with technology, attitude, and type of mental health concern.
  - Research available e-mental health tools to increase awareness and familiarity.
- Developers
  - Collaborate with Indigenous communities and organizations to ensure sensitive adaptation to different regions, communities, and languages.
  - Develop tools that combine practitioner support with client-led use.
  - Develop tools that monitor client outcomes in a clear, easy to interpret format.
  - Develop tools that support best practice through a structured approach and are appropriate for use by practitioners with little mental health training.
  - Ensure that the security of client data collected in tools matches the level of security in existing electronic information systems.
  - Develop tools that can be used without ongoing Internet connection.
  - Conduct research to expand the evidence base for effectiveness of e-mental health approaches.
  - Evaluate implementation strategies and practitioner fidelity to treatment.
  - Develop training packages that address gaps in expertise including cross cultural skills and familiarity with technology.
  - Work with service providers to promote awareness of e-mental health.
- Health Services
  - Assess the alignment of individual tools with existing information management systems and any needs for technical adaptations to existing systems prior to implementation. Internal directives are needed on how client data from tools are to be saved in client records.
  - Consider what investment may be needed in systems and supportive infrastructure including Internet connection, mobile/tablet devices and information systems prior to implementation.
  - Consider the need to adapt existing policies, procedures, and frameworks including clinical governance frameworks and policies on electronic equipment and storage and transmission of client data to take account of e-mental health.
  - Provide internal directives to advise which aspects of existing practice e-mental health tools will complement or replace; and guide the use of tools within or as an adjunct to client sessions.
  - Establish support structures to guide the use of e-mental health, such as supervision, regular review and troubleshooting support in order to help staff adapt to e-mental health approaches and to promote treatment fidelity.
  - Consult with staff and demonstrate support and commitment from senior managers through a “whole of organization” approach.
  - Develop implementation plans, which may include a consultation phase, a pilot process, a staged approach to introduction, integration with treatment pathways/usual practice, and establishing and reviewing implementation milestones.
  - Undertake a needs analysis of staff training, considering staff skills and experience in IT, counseling, and working cross-culturally. Training should be included in orientation processes to ensure sustainability of implementation efforts.
- Government
  - Develop training packages for e-mental health which include training in IT skills, counseling skills, and working cross-culturally and which are flexible for use in diverse contexts.
  - Promote awareness of e-mental health including strategies that lead to positive attitudes and motivation to use.
  - Invest further in supportive infrastructure such as Internet connection and faster download speeds in remote communities.
  - Work with service providers to ensure integration of the Digital Mental Health Gateway with diverse information management systems.
  - Make grants available to service providers for staff training and appropriate hardware for e-mental health.
  - Consider incentives for use of e-mental health approaches through the Medicare Benefits Schedule.

Our established relationships within the sector and our role as the developers of an e-mental health innovation may have influenced participant responses. Although these relationships may have enabled us to gain a deeper understanding of contextual factors, they also create potential for bias. Nevertheless, participant responses represent a full spectrum of views. The AIMhi Stay Strong App may have dominated participant perceptions about e-mental health; however, participant views about various features of that app are also relevant to the development of other e-mental health resources for Indigenous Australians. We have indicated above where participants explicitly or implicitly referred to the AIMhi Stay Strong App.

The format of qualitative interviews may have influenced participant responses, particularly where participant contributions were made in the presence of a line manager or other colleagues. However, participants were given the choice of taking part in a group or individual interview. These factors were taken into consideration in our analysis where there was potential for social influence in order to avoid overstating the numbers of participants sharing a single perspective.

### Conclusions

The present study contributes to understandings of the potential implementation of e-mental health approaches for Indigenous clients in health service settings. Results suggest e-mental health interventions may be most effective when used to support or extend existing health services, involving elements of client-driven and practitioner-supported use. Used in this context, e-mental health approaches offer more than simply a new modality of treatment and, if adopted, are likely to change the very nature of health care, impacting on access to care, the therapeutic relationship and data recording, in addition to influencing the functioning of health services.

As practitioners and service providers consider the opportunity presented by e-mental health, several client, practitioner, and system-level factors and the design of innovations are likely to influence the level of effectiveness, the degree of change needed to current practice and organizational processes and ultimately, the level of use. Developers, service providers, practitioners and government all have contributions to make in designing tools and related training that meet client and stakeholder needs, embedding e-mental health approaches in practice frameworks and systems and supporting appropriate use while seeking solutions to obstacles such as poor supportive infrastructure, financial constraints and workforce capacity issues.

## Appendix

Multimedia Appendix 1 Example of a practitioner-supported e-mental health tool.

## Abbreviations

ACCHO: Aboriginal community controlled health organization
CEO: chief executive officer
e-mental health: electronic mental health
eMHPrac: e-Mental Health in Practice
IT: information technology
NGO: non-government organization
