# Supplementary figures and images for: E-Mental Health Innovations for Aboriginal and Torres Strait Islander Australians: A Qualitative Study of Implementation Needs in Health Services
Source: JMIR Ment Health. 2016 Sep 19;3(3):e43. doi: 10.2196/mental.5837 (PMC5048059; doi:10.2196/mental.5837)

## Multimedia Appendix 1: Example of a practitioner-supported e-mental health tool

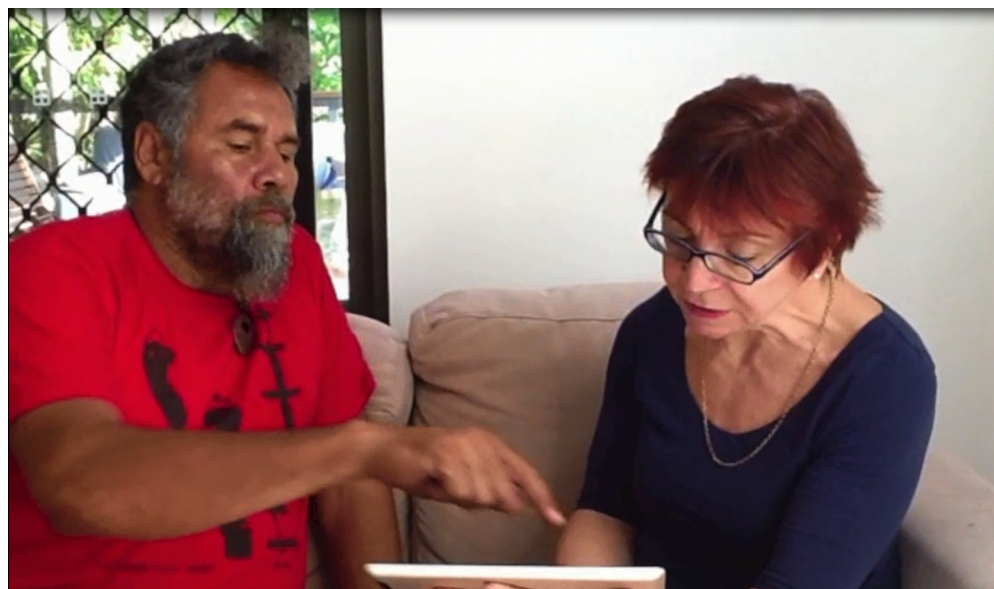

Supplement: Multimedia Appendix 1 [file mental_v3i3e43_app1.pdf]
